# The role of thyroid transcription factor-1 in differentiating lung adenocarcinomas from non-pulmonary adenocarcinoma effusions

**DOI:** 10.25122/jml-2023-0143

**Published:** 2023-06

**Authors:** Leena Sabir khidir, Mohamed Elfatih Abdelwadoud, Ammar Abdelmola, Nihad Elsadig Babiker, Ibrahim Bakhit Elemam, Osman Mohamed Elmahi, Nahed Sail Alharthi, Hisham Ali Waggiallah

**Affiliations:** 1 Department of Histopathology and Cytopathology, Faculty of Medical Laboratory Sciences, National University, Khartoum, Sudan; 2 Department of Histopathology and Cytopathology, Faculty of Medical Laboratory Sciences, University of Medical Sciences and Technology, Khartoum, Sudan; 3 Department of Medical Laboratory Sciences, Faculty of Applied Medical Sciences, Jazan University, Jazan, Saudi Arabia; 4 Department of Hematology, Faculty of Medical Laboratory Science, Darfur University College, Nyala, Sudan.; 5 Department of Histopathology and Cytopathology, Faculty of Medical Laboratory Sciences, Shendi University, Shendi, Sudan.; 6 Department of Histopathology and Cytopathology, Faculty of Medical Laboratory Sciences, Karary University, Omdurman, Sudan; 7 Department of Medical Laboratory Science, College of Applied Medical Science, Prince Sattam Bin Abdulaziz University, Saudi Arabia

**Keywords:** thyroid transcription factor-1, lung adenocarcinomas, non-pulmonary adenocarcinoma, effusions

## Abstract

Effusions, characterized by abnormal fluid accumulations in body cavities, present difficulties in identifying the primary organs of metastatic tumors through cytopathologic investigation, particularly in cancer-related complications. This retrospective cross-sectional laboratory study aimed to investigate the role of thyroid transcription factor-1 (TTF-1) in distinguishing lung adenocarcinomas from non-pulmonary adenocarcinomas in effusions. The study was conducted at Almobarak Cytopathology Laboratory, a private cytopathology laboratory. H&E was used to confirm the histological diagnosis of 58 archived cell blocks. TTF-1 immunostaining patterns were then correlated with the histological diagnosis. Statistical analysis, including numerical and graphical data summaries, was conducted using the Chi-square test in SPSS 23. TTF-1 expression was observed in 20 (34.4%) cases, while 38 (65.5%) cases showed no TTF-1 reaction. Positive TTF-1 was found in pleural fluid in 61.1 % of lung adenocarcinomas, while negative TTF-1 was found in only 3.4%. TTF-1 was not detected in the majority of peritoneal fluid samples. There was a highly significant relationship between pleural fluid, TTF-1, and lung adenocarcinoma (p=0.000). The data provided further evidence that TTF-1 is a useful marker for distinguishing pulmonary adenocarcinomas from non-pulmonary adenocarcinoma tumors.

## INTRODUCTION

Metastasis is the spread of cancerous cells from the initial location to other body parts, commonly occurring in body cavities such as the pleural, pericardial, and peritoneal [1]. Effusions are abnormal accumulations of fluids in body cavities attributed to cancer. Cytopathologic investigation of body fluids collected from serous cavities plays a crucial role in cytopathology [2] and is a relatively simple and noninvasive method. The cytopathologic investigation of effusions can recognize malignant cells. However, due to the similarity in morphology between primary and metastatic tumors, identifying the primary organs of metastatic tumors by cytopathologic examination alone is difficult. The need for a more powerful instrument to recognize the primary organs of metastatic tumors has expanded.

In the last two decades, immunohistochemical markers have played an important role in the differential diagnosis of malignant effusion cytology [3]. TTF-1 is a tissue-specific protein-containing transcription factor expressed preferentially in the thyroid and lung [4]. TTF-1 expression is restricted to bronchial and alveolar epithelial cells in normal lung tissue, essential for pulmonary function and morphogenesis.

TTF-1 has been observed in lung tumors in alveolar adenoma [5], adenocarcinoma [6], carcinoid tumor, large cell neuroendocrine carcinoma [7], and small cell carcinoma [7, 8]. TTF-1 expression in adenocarcinoma is particularly important in distinguishing pulmonary adenocarcinoma from non-pulmonary adenocarcinoma. Many studies in paraffin-embedded sections have recognized this function [1,9]. However, the role of TTF-1 in cytology preparations has rarely been recognized, and immunohistochemistry was used to investigate the expression of TTF-1 in malignant pleural and peritoneal effusion.

Adenocarcinoma is the most prevalent epithelial malignancy linked to body cavities fluids [10]. The lung, breast, and gastrointestinal tract are the most common primary sites of adenocarcinoma in malignant effusions [11]. Immunohistochemical (IHC) markers may help determine the primary site of malignancies, which is essential for prognostic and therapeutic purposes. There is a scarcity of studies demonstrating the utility of the TTF-1 marker in distinguishing lung adenocarcinoma from non-pulmonary adenocarcinoma on a cytology basis [4]. This study aimed to detect the TTF-1 marker in effusions as a differentiating marker between metastatic and primary malignancies.

## MATERIAL AND METHODS

This was a retrospective cross-sectional laboratory study conducted at Almobarak Cytopathology Laboratory, a private facility located in Khartoum. The laboratory receives approximately 10,000 samples annually, with effusions accounting for around 5% of the total samples. Data were extracted from medical records using a retrospective sampling technique, including sample serial number, age, and gender. The 58 archived cell blocks were divided into 12 lung adenocarcinoma samples, 8 breast adenocarcinoma samples, five malignant mesothelioma samples, two ovarian carcinoma samples, two liver carcinoma samples, one renal carcinoma sample, 4 GIT adenocarcinoma, and 24 samples of unknown origin. Two slides were sectioned from each block at a thickness of 5 microns. One slide was stained with Hematoxylin and Eosin (H&E) to confirm the histopathological diagnosis, while the other was stained with immunohistochemistry. The staining utilized a standard multilink detection kit (Dako detection kit Santa Clara, CA, USA) consisting of an endogenous peroxidase block, a nonspecific binding block, horse radish peroxidase, diaminobenzidine as a chromogen, and hematoxylin. The sections were immunostained using a primary polyclonal antibody against thyroid transcription factor-1 (TTF-1/clone 8G7G3/1, Clenovte, Cambridge, UK), with the staining reaction occurring in the nucleus.

The cytological diagnosis of all specimens was reported using a descriptive format, classifying them as benign or malignant and describing the cellular abnormality. The pathologist confirmed the malignant cases and their origins at the Almobarak Cytopathology laboratory, where the study was conducted. Histopathological immune markers (Cambridge, UK) and H&E stains were used for diagnosis, with normal thyroid tissue sections as positive controls.

## DATA ANALYSIS

Statistical analysis was conducted using SPSS (Statistical Package for the Social Sciences). The relationship between TTF-1 expression and study participants was investigated, and the chi-squared test was employed to assess the characteristics and type of cancer. Statistical significance was determined at a threshold of p≤0.5.

## RESULTS

### Characteristics of the study participants

A total of 58 cell blocks from patients with malignant effusions were included in this study. Among the study population, 31 (53.4%) were males, and 27 (46.5%) were females (Table 1). The average age of participants was 52 years. Among the age groups, 21-40 years had 24 patients (41.3%), 41-60 years had 21 patients (36.2%), and 61-80 years had 13 patients (22.4%) (Table 2). The geographical distribution of the patients showed that the majority came from the center (51.7%), followed by the north (27.5%), west (13.7%), and south (6.8%).

**Table 1:** Distribution of TTF-1 expression by gender

Gender	Frequency (%)	Positive TTF-1 (%)	Negative TTF-1 (%)	P-value
**Male**	31 (53.4)	12 (38.7)	19 (61.3)	0.32
**Female**	27 (46.5)	8 (29.6)	19 (70.4)	
**Total**	58 (100)	20 (34.5)	38 (65.5)	

**Table 2:** Distribution of TTF-1 expression among age groups

Age group	Frequency (%)	Positive TTF-1 (%)	Negative TTF-1 (%)	P-value
**21 – 40 years**	24 (41.3)	2	3	0.77
**41 – 60 years**	21 (36.2)	5	13	
**61 – 81 years**	13 (22.4)	5	8	
**Missing**		22 (37.9)		
**Total**	58 (100)	12 (20.7)	24 (41.3)	

Table 3 revealed that most of the 58 malignant effusions (81.0%) were plural, with the remainder being peritoneal effusions (18.9%). Table 4 shows the histopathology type of the 58 cases with malignant effusions. Lung adenocarcinoma constituted the highest percentage (12.6%), while renal carcinoma accounted for only 1.7% of the cases. The remaining 24 (41.3%) cases were of unknown origin and revealed a statistically significant (p=0.000) association between TTF-1 expression and cancer type. TTF-1 was present in 20 (34.4%) cases and had no reaction in 38 (65.5%). Positive TTF-1 was found in pleural fluid in lung adenocarcinoma (61.1%), while negative TTF-1 was found in only 3.4% of cases. TTF-1 was not found in most peritoneal fluid. As shown in Table 5, there was a significant correlation between pleural fluid, TTF-1, and lung adenocarcinoma (p=0.000).

**Table 3:** Distribution of effusion types in the study population (n=58)

Type	Frequency (%)
Plural effusions	47 (81)
Peritoneal effusions	11 (18.9)
Total	58 (100)

**Table 4:** Distribution of malignant cases by histopathology type and TTF-1 expression

Type	Frequency (%)	Positive TTF-1 (%)	Negative TTF-1 (%)	P-value
**Lung adenocarcinoma**	12 (20.6)	11 (91.7)	1 (8.3)	0.000*
**Breast adenocarcinoma**	8 (13.7)	0 (0.0)	8 (100)	
**Malignant mesothelioma**	5 (8.6)	0 (0.0)	5 (100)	
**GIT adenocarcinoma**	4 (6.8)	1 (25)	3 (75)	
**Ovarian carcinoma**	2 (3.4)	0 (0.0)	2 (100)	
**Liver carcinoma**	2 (3.4)	0 (0.0)	2 (100)	
**Renal carcinoma**	1 (1.7)	0 (0.0)	1 (100)	
**Unknown origin**	24 (41.3)	8 (33.3)	16 (66.7)	
**Total**	58 (100)	20	38	

* p≤0.5

**Table 5:** Presence of TTF-1 in different cancer types confirmed by histopathology technique in effusions

Effusion type	Pleural		Peritoneal	
	Positive TTF-1 N (%)	Negative TTF-1 N (%)	Positive TTF-1 N (%)	Negative TTF-1 N (%)
**Renal carcinoma**	0 (0.0)	1(3.4)	0 (0.0)	0 (0.0)
**Lung adenocarcinoma**	11 (61.1)	1(3.4)	0 (0.0)	0 (0.0)
**Breast adenocarcinoma**	0 (0.0)	8 (27.6)	0 (0.0)	0 (0.0)
**GIT adenocarcinoma**	0 (0.0)	1(3.4)	1(50)	2 (22.2)
**Malignant mesothelioma**	0 (0.0)	5(17.2)	0 (0.0)	0 (0.0)
**Ovarian carcinoma**	0 (0.0)	5 (17.2)	0 (0.0)	2 (22.2)
**Liver carcinoma**	0 (0.0)	2 (6.9)	0 (0.0)	0 (0.0)
**Unknown**	7 (38.9)	11 (37.9)	1 (50)	5 (55.6)
**Total**	18 (100)	29 (100)	2 (100)	9 (100)
**P-value**	0.000*		0.63	

* p≤0.5

**Figure 1:**
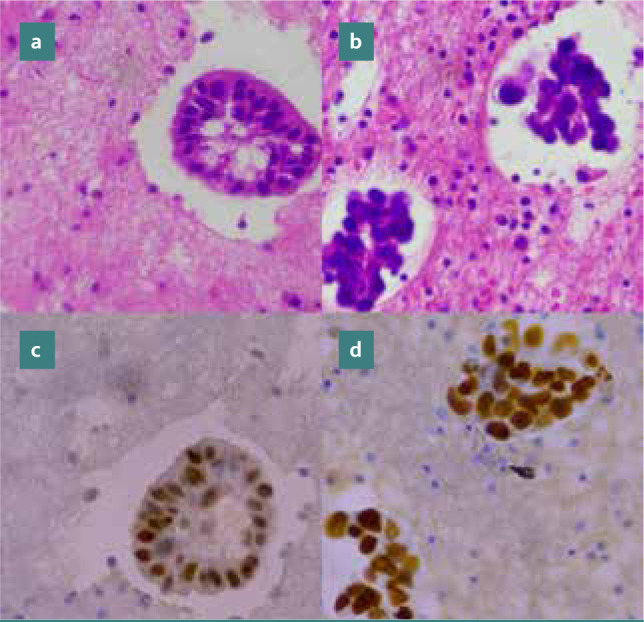
TTF-1 Immunoreactivity in Adenocarcinomas of Lung Origin in Pleural Effusions (Cell Block Preparations). (a-b) Hematoxylin and eosin stain showing adenocarcinomas of lung origin. (c-d) Magnification: x400, illustrating nuclear immunoreactivity for TTF-1.

**Figure 2:**
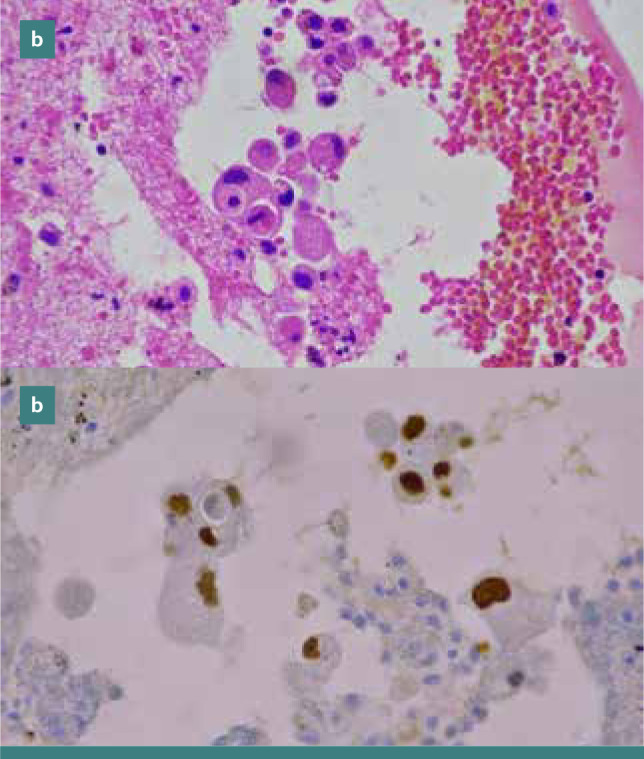
TTF-1 Immunoreactivity in Gastrointestinal Tract Adenocarcinomas in Peritoneal Effusions (Cell Block Preparations). (a) Hematoxylin and eosin stain showing nuclear immunoreactivity for TTF-1. (b) Magnification: x400.

Figure 1 (a-d) illustrates TTF-1 expressions in adenocarcinomas of lung origin in pleural effusions, while Figure 2 (a-b) shows TTF-1 expressions in gastrointestinal tract adenocarcinomas in peritoneal effusions.

## DISCUSSION

The distribution of demographic data among the participants in this study did not show a statistically significant association with TTF-1, consistent with the findings of Khoor *et al*. [4]. Furthermore, there was no significant relationship between gender and age with the TTF-1 expression in the study population.

All participants in this study had malignant effusions, with the majority (81.0%) having plural effusions and 18.9% having peritoneal effusions. The histopathological examination of 58 malignant effusions revealed that 24 samples (41.3%) were of unknown origin, which poses a diagnostic challenge for pathologists and, as a result, affects the therapeutic options for each patient. For the definitive diagnosis of an unknown origin sample, an immunohistochemical marker with high sensitivity and specificity is required.

The current study used tissue-specific markers in immunohistochemistry to subclassify adenocarcinomas based on their primary sites. TTF-1, a tissue-specific protein found in thyroid and lung epithelial cells, was tested for its ability to distinguish lung adenocarcinomas from other common adenocarcinomas in 58 body cavity fluid samples. The use of a commercial TTF-1 antibody product demonstrated positive TTF-1 expression in 11 out of 12 lung adenocarcinoma cases, while one non-pulmonary adenocarcinoma case showed positive TTF-1 expression. This resulted in a sensitivity of 91.6% and specificity of 98.1% for TTF-1 in lung adenocarcinoma. Similar findings were reported by Takano *et al*. [10], who observed a sensitivity of 83.3% and specificity of 97.9% for TTF-1 in distinguishing lung adenocarcinoma from non-pulmonary adenocarcinoma. Another similar result was observed in a meta-analysis that evaluated TTF-1 expression in metastatic lung carcinoma effusions in 20 studies, with 74.95% sensitivity and 99% specificity [12].

The present study used archival, cytocentrifuged cell blocks to determine TTF-1 immunoreactivity. One advantage of using these materials is that immunocytochemistry studies can be performed on the same cells used for diagnosis, providing an important useful tool even in fluids with low cellularity [2]. TTF-1 sensitivity in lung adenocarcinoma was slightly lower than previously reported sensitivity using paraffin-embedded material, which could be due to a reduction in intranuclear TTF-1 antigen exposure in the cytocentrifuged preparations. Conversely, the high specificity of TTF-1 in pulmonary adenocarcinoma in our study was comparable to that reported by others, suggesting that TTF-1 should be used as an essential immunocytochemical marker in body cavity fluid cytology [4-15].

The immunostaining pattern of TTF-1 is characterized by diffuse, granular, and strong brown nuclear staining. This distinct pattern allows for easy assessment of TTF-1 immunoreactivity, contrasting with the negative reaction observed in the nuclei of macrophages, lymphocytes, neutrophils, and mesothelial cells in the background.

TTF-1 immunoreactivity has been observed in only a small number of non-pulmonary adenocarcinomas [1]. One case of gastrointestinal tract adenocarcinoma was found in this study. Bejarano *et al*. [16] reported TTF-1 immunoreactivity in one of 66 gastric adenocarcinomas and one of eight endometrial adenocarcinomas. Klingen *et al*. [1] found TTF-1 immunoreactivity in 3% of 247 primary breast cancer tumors. Georg *et al*. [17] described the first case of malignant mesothelioma with thyroid transcription factor-1 immunohistochemical expression. However, TTF-1 was not detected in any of the malignant mesotheliomas studied by Khoor *et al*. [4].

Although TTF-1 had high sensitivity and specificity in this study and other studies [4-15], the definitive diagnosis of primary carcinoma sites in effusions requires a panel of immunohistochemical markers. Takano *et al*. [10] discovered that a panel of markers, including GATA-3, PAX-8, and TTF-1, helped identify or exclude common primary sites of carcinoma in effusions with high sensitivity and specificity. Another study by El Hag *et al*. [14] revealed that an IHC panel or dual stain of TTF-1 and Napsin-A could be more useful for identifying the roots of metastatic adenocarcinoma in serous effusions.

In this study, positive TTF-1 expression in lung adenocarcinoma was not associated with a better overall prognosis or patient survival. TTF-1 has been shown to be very beneficial as a marker of pulmonary adenocarcinoma diagnosis in our review. Previous studies have shown that TTF-1 positivity in lung adenocarcinoma tissues is a favorable prognostic marker [18]. Schilsky *et al*. [13] reported an association between TTF-1 expression and improved survival in patients with advanced lung adenocarcinomas.

Based on the findings and conclusions of this study, we recommend the incorporation of cell block preparation in effusion cytology samples using the traditional smear technique, along with the use of a panel of immunohistochemical markers, including TTF-1, for the definitive diagnosis of effusion cases.

## CONCLUSION

The results show that TTF-1 is a useful marker with high specificity and sensitivity for distinguishing pulmonary adenocarcinoma from non-pulmonary adenocarcinoma tumors in cell block preparations.

## Personal thanks

This publication was supported by the Deanship of Scientific Research at Prince Sattam bin Abdulaziz University.
